# Complete genome sequence of *Arcticibacterium luteifluviistationis* SM1504^T^, a cytophagaceae bacterium isolated from Arctic surface seawater

**DOI:** 10.1186/s40793-018-0335-x

**Published:** 2018-11-26

**Authors:** Yi Li, Xiao-Han Guo, Yan-Ru Dang, Lin-Lin Sun, Xi-Ying Zhang, Xiu-Lan Chen, Qi-Long Qin, Peng Wang

**Affiliations:** 1 State Key Laboratory of Microbial Technology, Marine Biotechnology Research Center, Shandong University, No.72, Binhai Rd, Qingdao, 266237 China; 2 Laboratory for Marine Biology and Biotechnology, Qingdao National Laboratory for Marine Science and Technology, No.1, Wenhai Rd, Qingdao, 266237 China

**Keywords:** *Arcticibacterium luteifluviistationis*, Secondary metabolite biosynthesis, Stress resistance, Carbohydrate metabolism, Arctic

## Abstract

*Arcticibacterium luteifluviistationis* SM1504^T^ was isolated from Arctic surface seawater and classified as a novel genus of the phylum *Bacteroides*. To date, no *Arcticibacterium* genomes have been reported, their genomic compositions and metabolic features are still unknown. Here, we reported the complete genome sequence of *A. luteifluviistationis* SM1504^T^, which comprises 5,379,839 bp with an average GC content of 37.20%. Genes related to various stress (such as radiation, osmosis and antibiotics) resistance and gene clusters coding for carotenoid and flexirubin biosynthesis were detected in the genome. Moreover, the genome contained a 245-kb genomic island and a 15-kb incomplete prophage region. A great percentage of proteins belonging to carbohydrate metabolism especially in regard to polysaccharides utilization were found. These related genes and metabolic characteristics revealed genetic basis for adapting to the diverse extreme Arctic environments. The genome sequence of *A. luteifluviistationis* SM1504^T^ also implied that the genus *Arcticibacterium* may act as a vital organic carbon matter decomposer in the Arctic seawater ecosystem.

## Introduction

As the third most abundant bacterial group in the seawater system, phylum *Bacteroidetes* plays a vital role in diverse oceanic biogeochemical processes [1]. It has been reported that phylum *Bacteroidetes* could mediate the degradation of HMW compounds especially in the respect of algal organic matter [2, 3]. Many heterotrophic microorganisms such as the SAR11 clade and marine *Gammaproteobacteria* grow partly due to phylum *Bacteroidetes*-derived organic products [4, 5]. Thus, phylum *Bacteroidetes* groups may play crucial roles in the nutrient utilization and cycling in the seawater ecosystem.

The family *Cytophagaceae*, currently comprising 31 genera, is one of the largest groups in the phylum *Bacteroidetes* [6]. The species in the family *Cytophagaceae* have been isolated from various habitats including freshwater river [7], seawater [8], permafrost soil [9] and even polar glacial till [10]. The genus *Arcticibacterium*, belonging to the family *Cytophagaceae*, accommodates only one recognized species: *A. luteifluviistationis* SM1504^T^ (=KCTC 42716^T^=CCTCC AB 2015348^T^) [11]. Strain SM1504^T^ was isolated from surface seawater of King’s Fjord, Arctic. However, to date, no genomes of the genus *Arcticibacterium* have been reported, their genomic compositions and metabolic pathways are still lacking. In the study, we reported the first genome sequence of the genus *Arcticibacterium* to better understand its survival strategy and ecological niche in the Arctic seawater.

## Organism information

### Classification and features

**Fig. 1:**
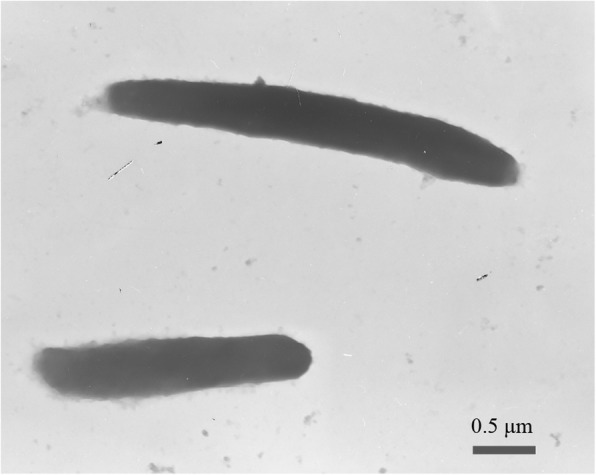
Transmission electron micrographs of *Arcticibacterium luteifluviistationis* SM1504^T^ cultured on TYS broth medium. Scale bar, 0.5 μm

**Fig. 2:**
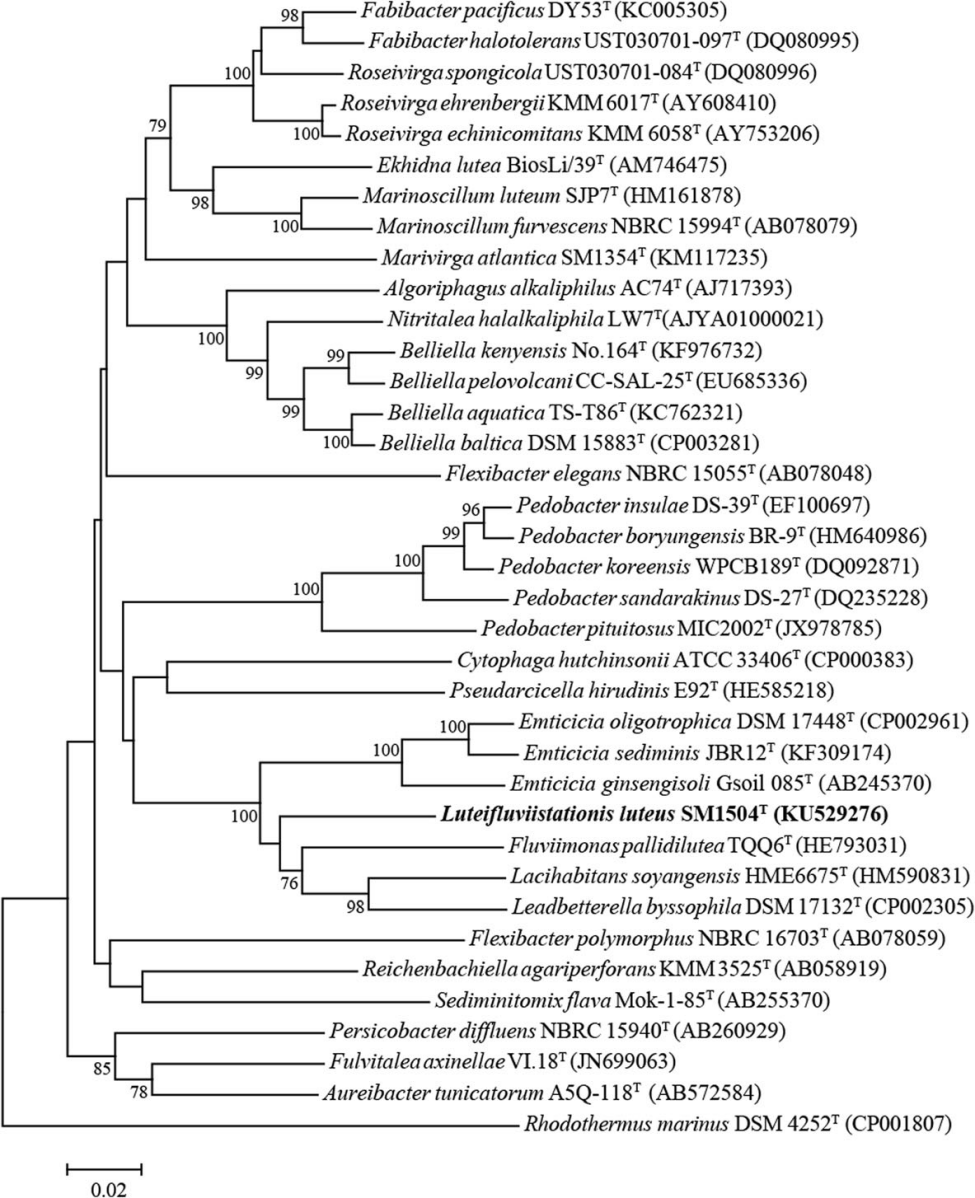
Neighbor-joining phylogenetic tree based on 16S rRNA gene sequences, showing the relationships of *Arcticibacterium luteifluviistationis* SM1504^T^ and its taxonomic neighbors. *Rhodothermus marinus* DSM 4252^T^ was used as as the outgroup. Bootstrap values (> 70%) based on 1000 replicates are shown at nodes. Bar, 0.02 substitutions per nucleotide position

As the type strain of *A. luteifluviistationis* in the family *Cytophagaceae*, strain SM1504^T^ is a Gram-negative, aerobic, non-motile and rod bacterium (Fig. 1). The yellow-pigmented colony was found after incubation at 20 °C for 2 days on a TYS agar plate. The strain could utilize glycerol, D-xylose, D-glucose, D-fructose, dulcitol, inositol D-mannitol, D-sorbitol, N-acetylglucosamine, arbutin, aesculin, cellobiose, maltose, sucrose, trehalose, starch, turanose and potassium gluconate for energy and growth, which were summarized in Table 1. Then it hydrolyzed aesculin, gelatin, tyrosine, Tween 20, 40 and 60 but did not hydrolyze DNA, agar, casein, elastin, lecithin, starch, Tween 80. In addition, various enzymes such as alkaline phosphatase, esterase (C4), esterase lipase (C8), leucine arylamidase, valine arylamidase, cystine arylamidase, trypsin and glucosidase were produced for degrading organic matter [11]. The phylogenetic placement of strain SM1504^T^ (based on complete 16S rRNA gene sequence) through neighbor-joining phylogenetic tree was identified (Fig. 2). It formed a distinct phylogenetic branch within the family *Cytophagaceae* and closely relatives were species of the genera *Lacihabitans*, *Emticicia*, *Fluviimonas* and *Leadbetterella* with low sequence similarities between 88.9 and 91.6%.

**Table 1:** Classification and general features of *Arcticibacterium luteifluviistationis* SM1504^T^ [12]

MIGS ID	Property	Term	Evidence code^a^
	Classification	Domain *Bacteria*	TAS [28]
		Phylum *Bacteroidetes*	TAS [29, 30]
		Class *Cytophagia*	TAS [30, 31]
		Order *Cytophagales*	TAS [32, 33]
		Family *Cytophagaceae*	TAS [32, 34]
		Genus *Arcticibacterium*	TAS [11]
		Species *Arcticibacterium luteifluviistationis*	TAS [11]
		Strain: *SM1504*^*T*^	TAS [11]
	Gram stain	Negative	TAS [11]
	Cell shape	Rod	TAS [11]
	Motility	Non-motile	TAS [11]
	Sporulation	Not reported	
	Temperature range	4–30 °C	TAS [11]
	Optimum temperature	20 °C	TAS [11]
	pH range; Optimum	6.0–7.5; 6.5–7.0	TAS [11]
	Carbon source	glycerol, D-xylose, D-glucose, D-fructose, dulcitol, inostiol D-mannitol, D-sorbitol, N-acetylglucosamine, arbutin, aesculin, cellobiose, maltose, sucrose, trehalose, starch, turanose and potassium gluconate	TAS [11]
MIGS-6	Habitat	seawater	TAS [11]
MIGS-6.3	Salinity	0–4% NaCl (*w*/*v*)	TAS [11]
MIGS-22	Oxygen requirement	Aerobic	TAS [11]
MIGS-15	Biotic relationship	Free-living	NAS
MIGS-14	Pathogenicity	Non-pathogen	NAS
MIGS-4	Geographic location	King’s Fjord, Arctic	TAS [11]
MIGS-5	Sample collection	2014	TAS [11]
MIGS-4.1	Latitude	Not reported	
MIGS-4.2	Longitude	Not reported	
MIGS-4.4	Altitude	Not reported	

^a^Evidence codes -*TAS* Traceable Author Statement, *NAS* Non-traceable Author Statement. These evidence codes are from the Gene Ontology project [35]

## Genome sequencing information

### Genome project history

Isolated from an extreme Arctic environment, *A. luteifluviistationis* SM1504^T^ was selected for genome sequencing to elucidate the special abilities of adapting to diverse extreme stresses. We have accomplished the genome sequencing of strain SM1504^T^ as reported in this paper. The complete genome data has been deposited in the GenBank database under the accession number CP029480.1. The project information and its association with MIGS are provided in Table 2 [12].

**Table 2:** Project information

MIGS ID	Property	Term
MIGS 31	Finishing quality	Complete
MIGS-28	Libraries used	Two genomic libraries: one Illumina library, one PacBio standard library
MIGS 29	Sequencing platforms	Illumina Hiseq 2500, PacBio RS
MIGS 31.2	Fold coverage	315× Illumina, 45× PacBio
MIGS 30	Assemblers	SOAPdenovo v. 2.04; HGAP v. 2.3.0
MIGS 32	Gene calling method	Prodigal
	Locus Tag	SM1504
	Genbank ID	CP029480.1
	GenBank Date of Release	June 20, 2018
	GOLD ID	Not registered
	BIOPROJECT	PRJNA471374
MIGS 13	Source Material Identifier	KCTC 42716^T^=CCTCC AB 2015348^T^
	Project relevance	Environmental, microbes

### Growth conditions and genomic DNA preparation

*A. luteifluviistationis* SM1504^T^ was cultivated in TYS broth at 20 °C. After cultivation for two days, genomic DNA for sequencing was extracted by using a commercial bacterial DNA isolation kit (OMEGA).

### Genome sequencing and assembly

Genome sequencing was performed on both the Illumina Hiseq and the PacBio RS sequencing platforms. 400-bp Illumina paired-end libraries and 20-kb PacBio libraries were constructed and sequenced yielding 315 × and 45 × average coverages, respectively (Table 2). About 1.69 Gb and 243 Mb data from the Illumina and PacBio sequencing were assembled using SOAPdenovo [13, 14] and HGAP [15]. The final assembly resulted in one scaffold.

### Genome annotation

Coding gene sequences were predicted and annotated through Prodigal v2.6.3 [16] and RAST v2.0 [17]. Functional categorization and carbohydrate-active enzymes CAZy of the predicted genes were annotated against EggNOG and CAZy databases, respectively. Then rRNAs and tRNAs were predicted by RNAmmer v1.2 [18] and tRNAscan-SE v1.3.1 [19]. In addition, the CARD analyses were performed to find resistance genes. Genomic islands and secondary metabolite biosynthesis were predicted through IslandViewer 4 [20] and antiSMASH [21].

## Genome properties

**Fig. 3:**
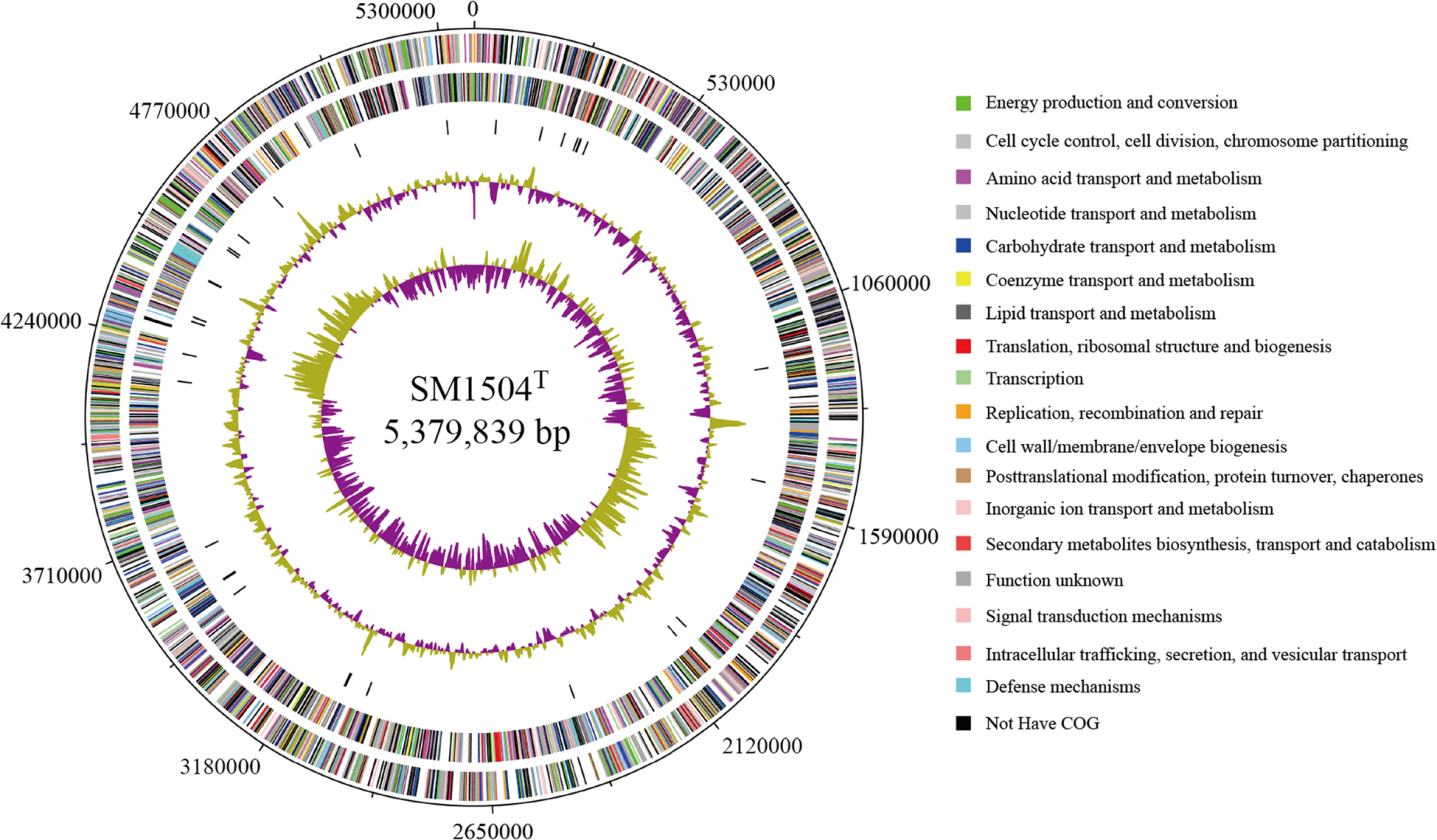
Circular map of the *Arcticibacterium luteifluviistationis* SM1504^T^ genome. From the outside to the center: CDSs on forward strand (colored by COG categories), CDSs on reverse strand (colored by COG categories), RNA genes (tRNAs and rRNAs), G + C content and GC skew

The total size of the genome of *A. luteifluviistationis* SM1504^T^ is 5,379,839 bp with an average GC content of 37.20% (Fig. 3). Total 4595 protein-coding genes (CDSs) were identified, which occupied 89.73% of the genome. Therein, 3045 CDSs were annotated with putative functions and 1550 CDSs matched hypothetical proteins (Table 3). Then 4 rRNAs and 36 tRNAs were found in the genome. CRISPR repeat, transmembrane helice, signal peptide and Pfam protein family predictions were done. In addition, distribution of genes into COG functional categories was shown in Table 4.

**Table 3:** Genome statistics

Attribute	Value	% of Total
Genome size (bp)	5,379,839	100
DNA coding (bp)	4,827,135	89.73
DNA G + C (bp)	2,029,275	37.20
DNA scaffolds	1	100.00
Total genes	4635	100.00
Protein coding genes	4595	99.14
RNA genes	40	0.86
Pseudo genes	0	0
Genes in internal clusters	NA	NA
Genes with function prediction	3045	65.70
Genes assigned to COGs	3319	71.61
Genes with Pfam domains	3617	78.04
Genes with signal peptides	693	14.95
Genes with transmembrane helices	988	21.32
CRISPR repeats	4	0.09

*NA,* not applicable

**Table 4:** Number of genes associated with general COG functional categories

Code	Value	%age	Description
J	148	3.19	Translation, ribosomal structure and biogenesis
A	0	0	RNA processing and modification
K	180	3.88	Transcription
L	121	2.61	Replication, recombination and repair
B	0	0	Chromatin structure and dynamics
D	17	0.37	Cell cycle control, Cell division, chromosome partitioning
V	68	1.47	Defense mechanisms
T	154	3.32	Signal transduction mechanisms
M	273	5.89	Cell wall/membrane biogenesis
N	3	0.06	Cell motility
U	29	0.63	Intracellular trafficking and secretion
O	129	2.78	Posttranslational modification, protein turnover, chaperones
C	201	4.34	Energy production and conversion
G	229	4.94	Carbohydrate transport and metabolism
E	211	4.55	Amino acid transport and metabolism
F	68	1.47	Nucleotide transport and metabolism
H	83	1.79	Coenzyme transport and metabolism
I	85	1.83	Lipid transport and metabolism
P	224	4.83	Inorganic ion transport and metabolism
Q	45	0.97	Secondary metabolites biosynthesis, transport and catabolism
R	0	0	General function prediction only
S	1080	23.30	Function unknown
–	1286	27.75	Not in COGs

The total is based on the total number of protein coding genes in the genome

## Insights from the genome sequence

### Adaption to diverse stresses

Strain SM1504^T^ genome owned two putative gene clusters for secondary metabolite biosynthesis. The cluster 1 belonged to terpene type - the largest group of natural products [22], matching the carotenoid biosynthesis. The cluster 2, affiliated to arylpolyene type, was predicted to produce flexirubin. Furthermore, we found that the yellow-pigmented strain SM1504^T^ harbors a complete set of genes required for zeaxanthin biosynthesis (e.g., isopentenyl-diphosphate delta-isomerase, phytoene synthase, phytoene dehydrogenase, lycopene cyclase and beta-carotene hydroxylase), which was commonly detected in other species of the phylum *Bacteroidetes* [23, 24]. The pigment maybe help the strain to obtain energy and for cold adaption and ultraviolet light protection in the Arctic environments [25].

A total of 150 resistance genes were found to encode 24 kinds of antibiotics (such as gentamicin, kanamycin, tetracycline and streptomycin), which was consistent with the experimental antibiotic susceptibility results [11]. The genes encoding heat shock proteins dnaK and cold shock protein cspA were detected in the genome. In line with this, SM1504^T^ had a wider growth temperature ranges (4–30 °C) [11]. Besides, the genome harbored several genes coding for catalase and superoxide dismutase to assist the strain at cellular and molecular levels in dealing harsh radiation in the Arctic. Dozens of genes related to osmotic stress (such as choline and betaine uptake and betaine biosynthesis) and carbon starvation responses were discovered in the *A. luteifluviistationis* genome, which would endow cells with tolerance to hyperhaline and oligotrophic environments.

As another feature, a 245-kb genomic island coding for 208 genes was predicted. Therein, 9 genes encoded proteins related to glucide biosynthesis, such aslipopolysaccharide core biosynthesis glycosyltransferase (lpsD), UDP-glucose dehydrogenase and capsular polysaccharide synthesis enzyme (Cap8C). In addition, the presence of transposases, integrases and mobile element proteins indicated that gene transfer has occurred in the *A. luteifluviistationis* SM1504^T^ genome [26]. Also, phage tail fiber proteins were predicted, which was in line with the analysis by PHAST [27] that a 15-kb incomplete prophage region could encode phage tail fiber proteins in the genome.

### Degradation and utilization of carbohydrates

Totally, 3319 (71.61%) genes could be assigned a COG function, of which the wall/membrane/envelope biogenesis (5.89%), carbohydrate transport and metabolism (4.94%) and inorganic ion transport and metabolism (4.83%) were enriched (Table 4). The high percentage of proteins related to carbohydrate transport and metabolism suggested that the strain SM1504^T^ could use various carbohydrates. On the other hand, the analyses from dbCAN showed that the strain SM1504^T^ possessed 341 genes which encoded carbohydrate metabolism enzymes, including 69 carbohydrate esterases (11 families), 125 glycoside hydrolases (46 families), 62 glycosyltransferases (22 families), 17 polysaccharide lyases (6 families), 12 auxiliary activities (3 families) and 56 carbohydrate-binding modules (15 families). Therein, a variety of enzymes are related to the degradation of macromolecular polysaccharides (e.g., xylanase, chitinase, mannanase, alpha amylase, endoglucanase, glucoamylase and alginate lyase) derived from marine macroalgae and phytoplankton. Those polysaccharases could hydrolyze a variety of macromolecular polysaccharides into small molecules that can be absorbed and metabolized by strain SM1504^T^ and other microorganisms in the seawater [4, 5].

## Conclusions

The genomic analyses showed that the strain SM1504^T^ could adapt to extreme Arctic seawater environments, such as high solar radiation, cold temperature and high salinity. Besides, it may act as a vital macromolecular polysaccharide decomposer and would play an important role in organic carbon cycling in the Arctic seawater ecosystem.

## Abbreviations

CARD: Comprehensive antibiotic resistance database
CAZy: Carbohydrate-active enzymes
CRISPR: Clustered regularly interspaced short palindromic repeats
HMW: High molecular weight
MIGS: Minimum information on the genome sequence
RAST: Rapid annotation using subsystem technology
TYS: Tryptone-yeast extract-sea salt
